# The Impact of cHS4 Insulators on DNA Transposon Vector Mobilization and Silencing in Retinal Pigment Epithelium Cells

**DOI:** 10.1371/journal.pone.0048421

**Published:** 2012-10-26

**Authors:** Nynne Sharma, Anne Kruse Hollensen, Rasmus O. Bak, Nicklas Heine Staunstrup, Lisbeth Dahl Schrøder, Jacob Giehm Mikkelsen

**Affiliations:** Department of Biomedicine, Aarhus University, Aarhus, Denmark; University of Birmingham, United Kingdom

## Abstract

DNA transposons have become important vectors for efficient non-viral integration of transgenes into genomic DNA. The *Sleeping Beauty* (SB), *piggyBac* (PB), and *Tol2* transposable elements have distinct biological properties and currently represent the most promising transposon systems for animal transgenesis and gene therapy. A potential obstacle, however, for persistent function of integrating vectors is transcriptional repression of the element and its genetic cargo. In this study we analyze the insulating effect of the 1.2-kb 5′-HS4 chicken β-globin (cHS4) insulator element in the context of SB, PB, and *Tol2* transposon vectors. By examining transgene expression from genomically inserted transposon vectors encoding a marker gene driven by a silencing-prone promoter, we detect variable levels of transcriptional silencing for the three transposon systems in retinal pigment epithelium cells. Notably, the PB system seems less vulnerable to silencing. Incorporation of cHS4 insulator sequences into the transposon vectors results in 2.2-fold and 1.5-fold increased transgene expression levels for insulated SB and PB vectors, respectively, but an improved persistency of expression was not obtained for insulated transgenes. Colony formation assays and quantitative excision assays unveil enhanced SB transposition efficiencies by the inclusion of the cHS4 element, resulting in a significant increase in the stable transfection rate for insulated SB transposon vectors in human cell lines. Our findings reveal a positive impact of cHS4 insulator inclusion for SB and PB vectors in terms of increased transgene expression levels and improved SB stable transfection rates, but also the lack of a long-term protective effect of the cHS4 insulator against progressive transgene silencing in retinal pigment epithelium cells.

## Introduction

DNA transposons are mobile DNA elements with a natural ability to integrate genetic material into genomic DNA. Consisting of only two parts, a transposon element defined by inverted terminal repeat sequences and a transposase enzyme mediating excision and reintegration of the transposon element, DNA transposons can easily be transformed into plasmid-based gene vector systems. Transposons have long been used for gene transfer applications in invertebrate model organisms, such as *Drosophila* and *Caenorhabditis elegans* [1], [2], but elements with efficient transposition in mammalian cells have been in high demand for biomedical and therapeutic applications. Reconstruction of *Sleeping Beauty* (SB), a *Tc1/mariner* element assembled from inactive salmonid fish transposon sequences, revealed the first DNA transposon vector reported to have high activity in vertebrate cells [3]. Since its resurrection, the SB system has proven to be active in a wide range of vertebrate species, which has made it a widely used non-viral tool for transgenesis and insertional mutagenesis studies [4], [5]. In addition, observations of long-term gene expression after SB-mediated delivery in human primary cell types (including CD34^+^ [6], [7], [8], primary T [9], [10], [11], [12], and embryonic stem cells [13], [14]), have made the SB transposon a highly studied vector system for gene therapy applications [15], [16]. In consequence, the first clinical trial utilizing SB-directed gene insertion has recently been initiated for adoptive immunotherapy treatment of patients with B-cell malignancies [17].

Since the re-activation of the SB transposon other transposable elements, capable of high-efficient transposition in mammalian cells, have been discovered. Amongst these are two naturally active elements, the piggyBac (PB) transposon, originally isolated from the cabbage looper moth *Trichoplusia ni* [18], [19], and the *Tol2* transposon, isolated from the genome of the Japanese medaka fish *Oryzias latipes* [20]. PB, the founding member of the *piggyBac* transposon family, has high transposition activity in numerous invertebrate organisms [21], and is highly active in mouse and human cells [22], [23], [24], including therapeutically relevant cells such as human embryonic stem cells [25] and human primary T cells [26], [27], [28], [29]. The PB transposon has also been shown to be a suitable vector system for reprogramming of human and mouse fibroblasts to induced pluripotent stem cells [30]. The *Tol2* element, a member of the *hAT* transposon family, is the favored transposon system for transgenesis and insertional mutagenesis studies in zebrafish ([31]). In addition, the *Tol2* transposon system has been shown to be active, although with lower efficiency than SB and PB, in all vertebrate cells tested so far [32].

The SB, PB and *Tol2* transposons originate from different phylogenetic backgrounds and possess different biological properties that may represent strengths or weaknesses in a vector context. Understanding functional differences contributes to an improved basis for choosing the most suited transposon vector system for a particular experimental or therapeutic application. Such differences include, first of all, the way of transposition. Excision of the SB transposon results in 2 or 3 bp 3′-overhangs at the transposon ends, and repair of the 3′-overhang creates, together with the TA target site duplication, a characteristic transposition footprint [33]. Excision of PB, in contrast, results in hairpin formations at the excised transposon ends, and the 5′-TTAA overhangs created in the flanking DNA after excision anneal in the absence of DNA synthesis, leaving an intact excision site without any transposition footprint [34]. *Tol2* also forms hairpin structures during transposition, but the hairpins are formed at the ends of the flanking donor DNA instead of at the ends of the excised element, and footprints of variable lengths are created at the excision site [35]. Secondly, SB, PB and *Tol2* differ in their cargo-capacity. Whereas SB vectors have been observed to have a reduced transposition activity with increased transposon size [36], [37], PB and *Tol2* seem less affected by cargo size and may carry up to at least 10-kb sequences without a significant reduction in transposition activity [24], [38]. Notably, the PB transposase was recently reported to mobilize a 100-kb transposon in mouse embryonic stem cells [39]. Thirdly, the integration site preferences of the three transposon systems vary. At the primary DNA sequence level, the SB and PB transposons are very strict in their choice of target site (TA dinucleotide or TTAA tetranucleotide, respectively), whereas the *Tol2* transposon is less discriminating by targeting typically an 8-bp sequence that can vary in nucleotide composition. At the genomic level, SB has a fairly random integration profile with no preference for or against genes [40], [41], [42], [43]. PB, in contrast, exhibits a nonrandom integration pattern with about 50% integration events within intragenic regions (dependent on the cell type) and a bias towards transcriptional start sites [23], [28], [42], [43], [44] and actively transcribed genes [28]. In case of *Tol2*, the integration pattern seems to vary depending on cell type with reports of less than 40% to almost 50% of the insertion events within transcriptional units and with a preference for integration near transcriptional start sites [43], [44], [45], [46].

Transcriptional repression of transgene cassettes constitutes a potential problem for transgenesis and therapeutic gene transfer applications. Recombinant retroviruses are highly susceptible to transcriptional silencing and position effects conducted by chromosomal sequences at the integration sites [47], [48], [49]. We have previously detected transcriptional silencing of a SB transposon-delivered transgene cassette in HeLa and F9 murine teratocarcinoma cells [50], [51]. By analyzing gene expression from F9 clones harboring SB transposons encoding a reporter gene driven by an RSV promoter, we observed that approximately 50% of the clones were silenced after 7 weeks of passage under non-selective conditions. Thus, SB does not seem to contain natural insulator elements that protect its content from pathways of transcriptional silencing. Such insulators are *cis*-acting elements that can block enhancer-activation of a promoter and/or protect transcribed regions from chromosomal position effects, offering protection against spreading of condensed inactive chromatin. The 5′-HS4 chicken β-globin (cHS4) insulator, a 1.2-kb sequence located at the 5′ end of the chicken β-globin domain, possesses both enhancer- and barrier-blocking effects, the latter which are coupled to hyperacetylation of histones H3 and H4 as well as a reduction in promoter CpG methylation [52]. Incorporation of cHS4 sequences into retroviral vectors improves expression of integrated transgene cassettes [53], [54] and provides increased and consistent expression of therapeutic transgenes delivered by lentiviral vectors [55]. The incorporation of cHS4 sequences into SB DNA transposon vectors also has positive influence on the stability of transgene expression in embryonic cells [51].

Cells of the retina pigment epithelium (RPE) are target cells of ongoing gene therapy trials [56], [57]. A spontaneously arising human RPE cell line, ARPE-19, has functional properties of RPE cells *in vivo* [58] and has been used frequently as an *in vitro* platform for molecular and genetic studies of retinal pigment epithelium. Recently, genetically engineered ARPE-19 cells were explored as a cellular source of nerve growth factor (NGF). ARPE-19 cells were encapsulated in a biodelivery device that was implanted in the basal forebrain of minipigs, facilitating *in vivo* delivery of NGF [59]. The therapeutic potential of this approach was recently further supported by the production of an improved ARPE-19-derived cell line in which NGF was expressed from a total of four integrated SB DNA transposon vectors encoding NGF [60]. In the present study, we further investigated long-term expression of DNA transposon-directed transgene expression in ARPE-19 cells with the goal of investigating transcriptional repression in these cells and compare cHS4-mediated insulation of expression cassettes contained within SB, PB, and *Tol2* transposon vectors. By analyzing eGFP expression driven by the silencing-prone RSV promoter in stably transfected ARPE-19 cell clones, we observed progressive transgene silencing as expected but notably detected variable levels of transgene silencing for the three transposon systems. Under these conditions, the PB system was least affected by transcriptional repression. Although cHS4 insulator sequences flanking the transgene cassette were found to protect against early position effects, resulting in increased initial transgene expression, our data also demonstrate that cHS4 elements do not offer long-term protection against repression in ARPE-19 cells, and shut-down of expression was seen even in clones with multiple transposon insertions. Interestingly, our findings demonstrate that incorporation of cHS4 sequences is beneficial for mobilizing SB transposons from plasmid DNA supporting an improved stable transfection rate.

## Results

### Efficient SB, PB and Tol2 transposition in ARPE-19 cells

**Figure 1:**
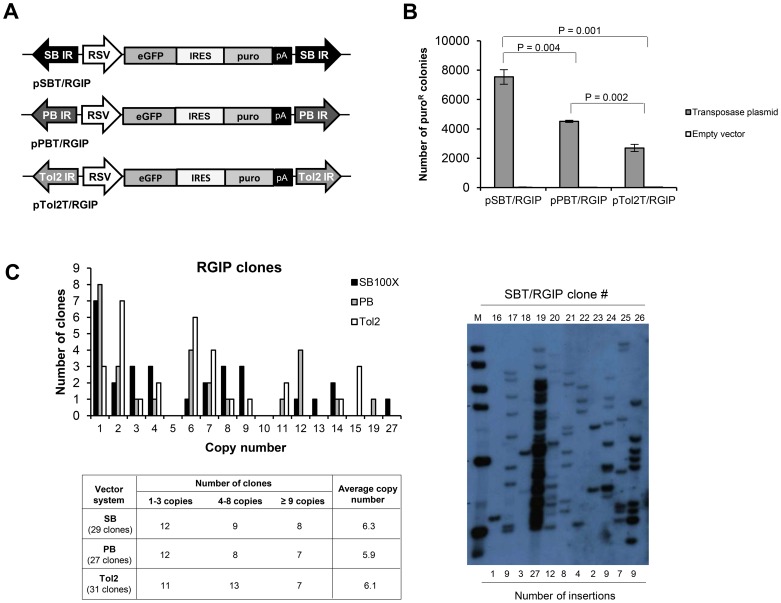
Transposition of SB, PB, and *Tol2* transposon vectors in ARPE-19 cells. (**A**) Schematic representation of pSBT/RGIP, pPBT/RGIP, and pTol2T/RGIP vectors. IR, inverted repeat; RSV, Rous sarcoma virus promoter; eGFP, enhanced green fluorescent protein; IRES, internal ribosome entry site; puro, puromycin resistance gene; pA, polyadenylation site. (**B**) Stable transfection rates of SB, PB, and *Tol2* transposon vectors in ARPE-19 cells. 0.125 pmol of pSBT/RGIP, pPBT/RGIP, and pTol2T/RGIP plasmid were cotransfected together with 0.02 pmol pcDNA3.1D/V5.TOPO plasmid (empty vector) or 0.02 pmol helper plasmid expressing either *SB100X* transposase, *iPB* transposase, or *Tol2* transposase. The pcDNA3.1D/V5.TOPO plasmid was also included as non-specific DNA to ensure that the total amount of DNA was 1 µg in each transfection. After 8 days of selection, puromycin resistant colonies were stained and counted. Mean ± SEM values are shown (N = 3). P values listed above the brackets were obtained by student's t-tests. (**C**) Transposon copy number of stably transfected ARPE-19 clones. Genomic DNA from ARPE-19 cell clones carrying SBT/RGIP, PBT/RGIP, or Tol2T/RGIP transposons was purified and examined by Southern blot analysis to determine the transposon copy number. A representative Southern blot is shown.

To study mobilization and transgene expression levels of DNA transposon-derived vectors in ARPE-19 cells, we generated vectors in the context of SB, PB, and *Tol2*, containing an RSV-driven bicistronic gene cassette consisting of the eGFP reporter gene, an internal ribosomal entry site (IRES), and the puromycin resistance gene (puro) [51]. The vectors were designated pSBT/RGIP, pPBT/RGIP, and pTol2T/RGIP, respectively (**Figure 1A**). Transposition efficiencies of the three vectors in ARPE-19 cells were measured by standard colony formation assays. Equal molar amounts of pSBT/RGIP, pPBT/RGIP, and pTol2T/RGIP were transfected together with pcDNA3.1D/V5.TOPO plasmid (empty vector) or a helper plasmid expressing either SB100X transposase [7], the native iPB transposase [24], or *Tol2* transposase [61]. This set of transposase variants were not chosen with the intention of directly comparing transposition capabilities but with the expectation of generating series of stable cell clones with a comparable number of insertions. After 8 days of selection, puromycin-resistant colonies were stained and counted. As shown in **Figure 1B**, the three combinations of transposon vectors and transposase promoted variable levels of stable transfection, with the SBT/RGIP transposon mobilized with SB100X showing a stable transfection rate that was 1.7-fold and 2.8-fold higher than the rates of PBT/RGIP (mobilized with iPB transposase) and Tol2T/RGIP (mobilized with the *Tol2* transposase), respectively (p<0.01). A more than 100-fold difference in colony formation between transfections with transposase plasmid and transfections with empty vector was observed for each transposon system, indicating that all three vectors were efficiently transposed in ARPE-19 cells.

Next, we constructed three series of ARPE-19 clones containing genomic insertions of the SBT/RGIP, PBT/RGIP, or Tol2T/RGIP transposons, respectively. Equal molar amounts of pSBT/RGIP, pPBT/RGIP or pTol2T/RGIP were transfected together with SB100X, iPB, or *Tol2* transposase-expressing plasmid, respectively, into ARPE-19 cells, and single colonies were subsequently isolated after puromycin selection. Three groups of 29 SB clones, 27 PB clones and 31 *Tol2* clones were isolated in total. Southern blot analysis of genomic DNA from all expanded cell clones (representative Southern blot shown in **Figure 1C**) revealed a large variation in the number of transposon insertions per clone within each group. In all three groups, a variation in transposon copy number ranging from 1 copy per clone to more than 10 copies per clone was observed (**Figure 1C**). However, the average copy number was comparable for all three groups, with 6.3 copies per clone for SB clones, 5.9 copies per clone for PB clones and 6.1 copies per clone for *Tol2* clones. Hence, the small difference in transposition activity obtained with the three transposase variants did not translate into a substantial difference in the transposon copy number.

### Postintegrative silencing of RGIP-containing ARPE-19 clones – the PB vector seems less vulnerable to transcriptional repression compared to SB and Tol2

**Figure 2:**
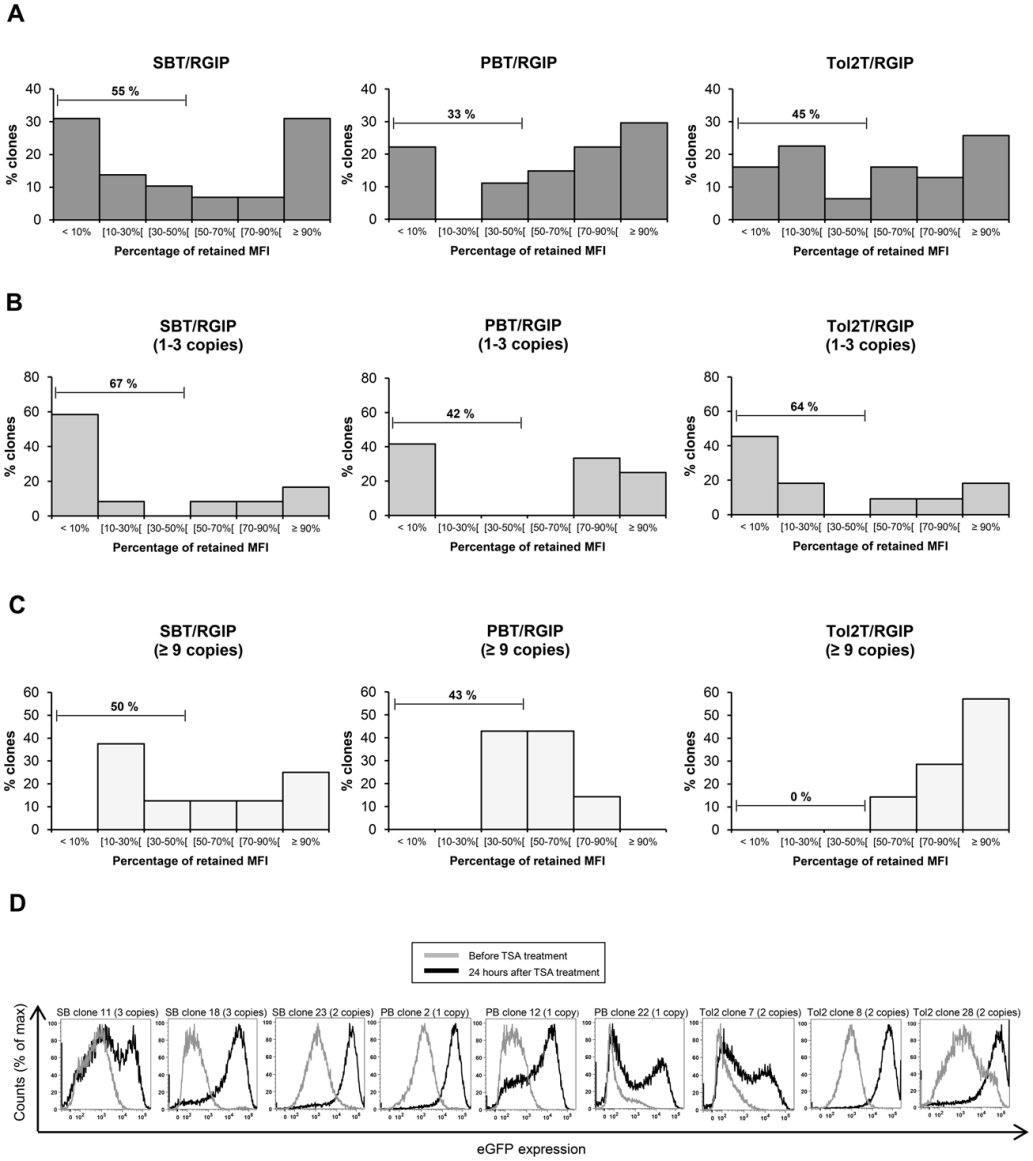
Silencing of SB, PB, and *Tol2* transposon-based vectors in ARPE-19 cells. (**A**) Percentage of retained median fluorescence intensity (MFI) for stably transfected ARPE-19 clones. ARPE-19 cells were transfected with pSBT/RGIP, pPBT/RGIP, or pTol2T/RGIP together with a transposase expressing plasmid, and selected for puromycin resistance. Individual clones were expanded and then passaged for 8 weeks in the absence of selection. Their eGFP expression level was determined at day 0 and day 56 of passage, and their percentage of retained MFI was calculated. (**B**) Percentage of retained median fluorescence intensity (MFI) for stably transfected ARPE-19 clones containing 1–3 transposon insertions. (**C**) Percentage of retained median fluorescence intensity (MFI) for stably transfected ARPE-19 clones containing 9 or more transposon insertions. (**D**) Reactivation of eGFP expression by TSA treatment. A subset of silenced ARPE-19 cell clones was grown in the presence of the deacetylase inhibitor Trichostatin A (TSA). The clones were treated 24 hours before analysis of eGFP expression by flow cytometry.

To compare the persistency of gene expression from genomically inserted SB, PB and *Tol2* vectors, the isolated clones were passaged for 8 weeks in the absence of puromycin, and eGFP expression levels were measured by flow cytometry at day 0 and day 56 of passage. Expression profile overlays are shown in **Supplementary Figures S1 to S3**. A reduction in transgene expression after 56 days of passage was observed for cell clones derived from all three transposon systems, but the level of reduction varied between the three vectors. For PB vector insertions, 33% of the clones had lost more than 50 percent of their initial expression, whereas 45% and 55% of the clones containing *Tol2* and SB transposons, respectively, had lost more than half of the initial expression (**Figure 2A**). A statistical comparison of SB and PB datasets, by the nonparametric Mood's median test, revealed a p-value of 0.061, showing that the decreased level of transgene repression observed for clones carrying the PB vector compared to clones containing the SB vector, was close to being statistical significant. When considering the clones with a low transposon copy number ranging from 1 to 3 copies per clone, SB and *Tol2* showed comparable levels of transgene silencing with 67% of the SB clones and 64% of the *Tol2* clones having lost more than 50 percent of their initial eGFP expression (**Figure 2B**). For the set of PB clones, 42%, of the clones expressed less than half of the initial eGFP expression, suggesting again that PB insertions carrying the RSV-driven gene cassette were less vulnerable to silencing. Notably, even ARPE-19 clones containing 9 or more transposon insertions were transcriptionally silenced over time (**Figure 2C**). Of the 8 SB clones containing 9 or more transposon copies, 38% had lost more than 70 percent of their initial transgene expression, and half of the clones had lost more than 50 percent of their initial expression. Of the 7 PB clones, 42% had lost more than half of their initial expression, but none of these clones had lost more than 70 percent of their initial expression. Notably, *Tol2* clones with 9 or more transposon copies had a modest reduction in transgene expression, with all 7 clones keeping more than 50% of their initial eGFP expression, indicating that expression was retained by *Tol2* in high copy clones.

To verify that the reduction in transgene expression observed for each transposon system was a result of epigenetic mechanisms and not a consequence of other factors such as loss of genetic material, we treated a subset of clones from each group with the histone deacetylase inhibitor TSA. Flow cytometric analysis 24 hours after TSA treatment showed that addition of TSA could fully or partially restore transgene expression levels for all treated clones (**Figure 2D**). Together our data indicate that genomically integrated RSV-driven eGFP transgene cassettes were subjected to transcriptional silencing in the context of all three transposon vector systems. This is expected, as the RSV promoter is known to be prone to transcriptional silencing. Nevertheless, the degree of transcriptional repression varied between the systems, suggesting that the PB system in the context of ARPE-19 cells was overall less vulnerable to transcriptional repression.

### Increased transgene expression levels from insulated SB and PB transposon vectors, but limited long-term protection against silencing by cHS4 insulators in ARPE-19 cells

**Figure 3:**
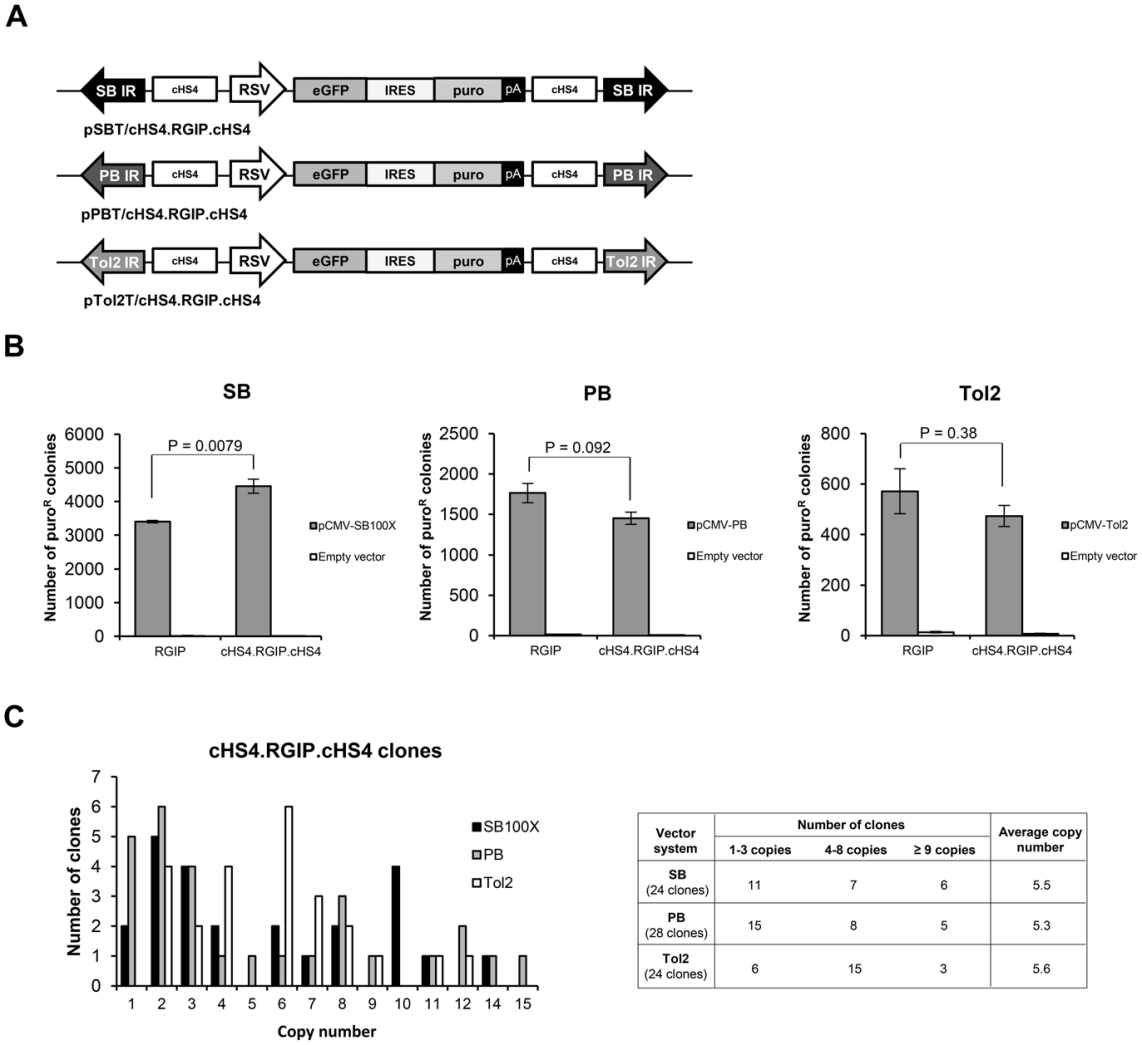
Transposition of insulated SB, PB, and *Tol2* transposon vectors in ARPE-19 cells. (**A**) Schematic representation of pSBT/cHS4.RGIP.cHS4, pPBT/cHS4.RGIP.cHS4, and pTol2T/cHS4.RGIP.cHS4 vectors. IR, inverted repeat; cHS4, 5′-HS4 chicken β-globin insulator sequence; RSV, Rous sarcoma virus promoter; eGFP, enhanced green fluorescent protein; IRES, internal ribosome entry site; puro, puromycin resistance gene; pA, polyadenylation site. (**B**) Stable transfection rates of SB, PB, and *Tol2* transposon vectors in ARPE-19 cells. 0.125 pmol transposon plasmid was transfected together with 0.05 µg transposase plasmid or pcDNA3.1D/V5.TOPO plasmid (empty vector) into ARPE-19 cells. The pcDNA3.1D/V5.TOPO plasmid was also included as non-specific DNA to ensure that the total amount of DNA was 1 µg for each transfection. After 8 days of selection, puromycin-resistant colonies were stained and counted. Mean ± SEM values are shown (N = 3). P values listed above the brackets were obtained by student's t-tests. (**C**) Transposon copy number of stably transfected ARPE-19 clones containing insulated transposon vectors. Genomic DNA from ARPE-19 cell clones carrying SBT/cHS4.RGIP.cHS4, PBT/cHS4.RGIP.cHS4, or Tol2T/cHS4.RGIP.cHS4 transposons was purified and examined by Southern blot analysis to determine the transposon copy number.

We have previously observed that cHS4 insulators flanking the RSV-GIP transgene cassette in the context of an SB vector leads to protection of the genomically inserted transgene against silencing in embryonic carcinoma cells [51]. To investigate the effect of cHS4 insulators in all three vector systems, we inserted two transgene-flanking cHS4 sequences in each of the vectors resulting in the plasmids pSBT/cHS4.RGIP.cHS4, pPBT/cHS4.RGIP.cHS4, and pTol2T/cHS4.RGIP.cHS4 (**Figure 3A**). The stable transfection rate of the insulated transposon vectors in ARPE-19 cells was analyzed by colony formation assays. As shown in **Figure 3B**, a significant 1.3-fold increase in the number of puromycin-resistant colonies was observed for transfections with SBT/cHS4.RGIP.cHS4 relative to transfections with SBT/RGIP (p = 0.0079) despite the increased size of the transposon vector. A similar positive effect of the cHS4 insulator on the stable transfection rate could not be observed for PB and *Tol2* vectors (**Figure 3B**). To generate ARPE-19 clones with insulated transposon vectors, groups of ARPE-19 clones containing SBT/cHS4.RGIP.cHS4 (total of 24 clones), PBT/cHS4.RGIP.cHS4 (total of 28 clones), and Tol2T/cHS4.RGIP.cHS4 (total of 24 clones), respectively, were isolated and expanded. The average number of transposon insertions (determined by Southern blot analysis; not shown) was 5.5, 5.3, and 5.6 copies per clone in the three cHS4-insulated transposon groups, respectively (**Figure 3C**), indicating that the insulated transposon vectors gave rise to a slightly lower average transposon copy number compared to uninsulated vectors.

**Figure 4:**
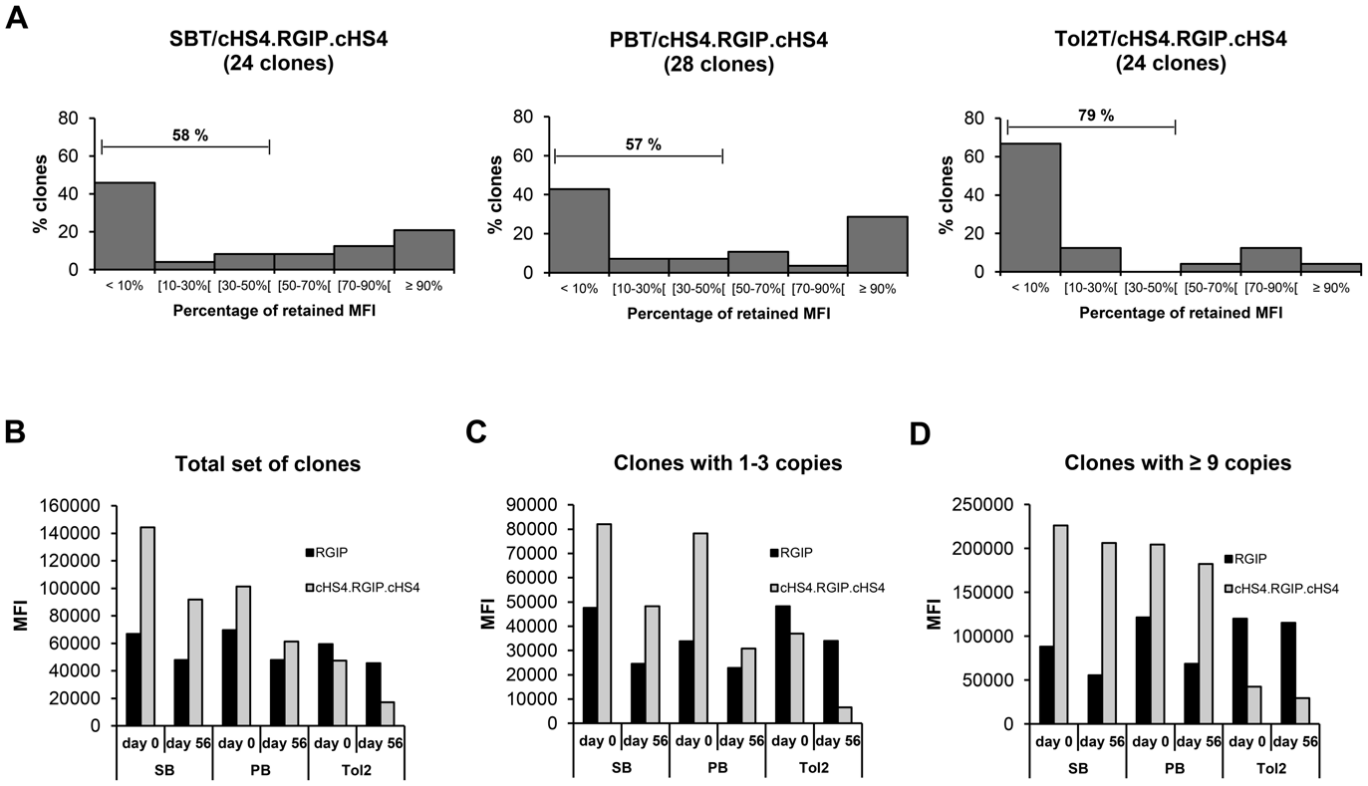
Insulation of SB, PB, and Tol2 transposon vectors in ARPE-19 cells. (**A**) Percentage of retained median fluorescence intensity (MFI) for stably transfected ARPE-19 clones carrying insulated transposon vectors. Measurements were obtained as described in Figure 2a. (**B**) Comparison of mean MFI levels for insulated and uninsulated clones. Stably transfected ARPE-19 cell clones were grown for 8 weeks in the absence of selection, and eGFP expression levels were measured by flow cytometry at day 0 and day 56 of passage. (**C**) Comparison of mean MFI levels for insulated and uninsulated clones carrying 1-3 transposon insertions. (**D**) Comparison of mean MFI levels for insulated and uninsulated clones carrying 9 or more transposon insertions.

To study possible protective effects of the cHS4 element in SB, PB and *Tol2* vectors in ARPE-19 cells, clones containing insulated transposon vectors were passaged for 8 weeks under non-selective conditions. The eGFP expression level was measured for each clone by flow cytometry at day 0 and day 56 of passage (**Supplementary Figures S4 to S6**). Notably, we did not observe a long-term protective effect of the cHS4 element against transcriptional silencing of the RSV-GIP transgene cassette (**Figure 4A**). Rather, a large portion of the clones carrying insulated transposon vectors were subjected to transcriptional repression. Insulated *Tol2* clones were most affected, with 79% of the clones having lost more than 50 percent of their initial expression compared to 58% and 57% for SB- and PB-containing clones, respectively (**Figure 4A**). TSA treatment of a subset of clones carrying insulated vectors showed that expression levels could be restored for all treated clones (data not shown), indicating again that epigenetic modifications were responsible for the decrease in transgene expression.

To determine if the cHS4 insulator had an effect on transgene expression levels, we examined the median fluorescence intensity (MFI) level for clones with and without cHS4 sequences. In each group of ARPE-19 clones, large variations in MFI were observed, even for clones with an equal copy number (**Supplementary Figure S7**), suggesting that the chromosomal environment at the insertion site had a substantial impact on eGFP expression due to position effects. However, the mean values of MFI were increased 2.2-fold and 1.5-fold for clones carrying insulated SB and PB vectors, respectively, compared to clones with uninsulated vectors at day 0 of passage (**Figure 4B**). At day 56 of passage, this relative increase in mean MFI was 1.9-fold for insulated SB clones and 1.3-fold for insulated PB clones. Since the average transposon copy number was slightly lower for insulated clones compared to uninsulated clones, the result suggests that incorporation of cHS4 sequences into the SB and PB vectors could lead to increased gene expression levels from the transgene cassette. Similarly, insulated SB and PB clones containing 1 to 3 transposon insertions exhibited between 1.4 and 2.3-fold increased expression levels (**Figure 4C**). However, the positive effect of the cHS4 insulator on transgene expression levels was most pronounced in clones with a high copy number, where the increase in mean MFI was observed to be as high as 3.7-fold for SB clones and 2.7-fold for PB clones at day 56 of passage (**Figure 4D**). In *Tol2* clones, mean MFI values were consistently lower for clones carrying insulated transposons compared to clones containing uninsulated transposons, suggesting that incorporation of cHS4 sequences into the *Tol2* vector did not lead to a protection against repressive position effects. In summary, these results indicate that the cHS4 insulator did not have a long-term stabilizing effect on transgene expression from SB, PB, and *Tol2* vectors in ARPE-19 cells, but that inclusion of cHS4 sequences had an overall beneficial effect on the level of transgene expression from integrated SB and PB transposon vectors.

### Increased transposition of insulated SB transposon vectors in ARPE-19 and HeLa cells

**Figure 5:**
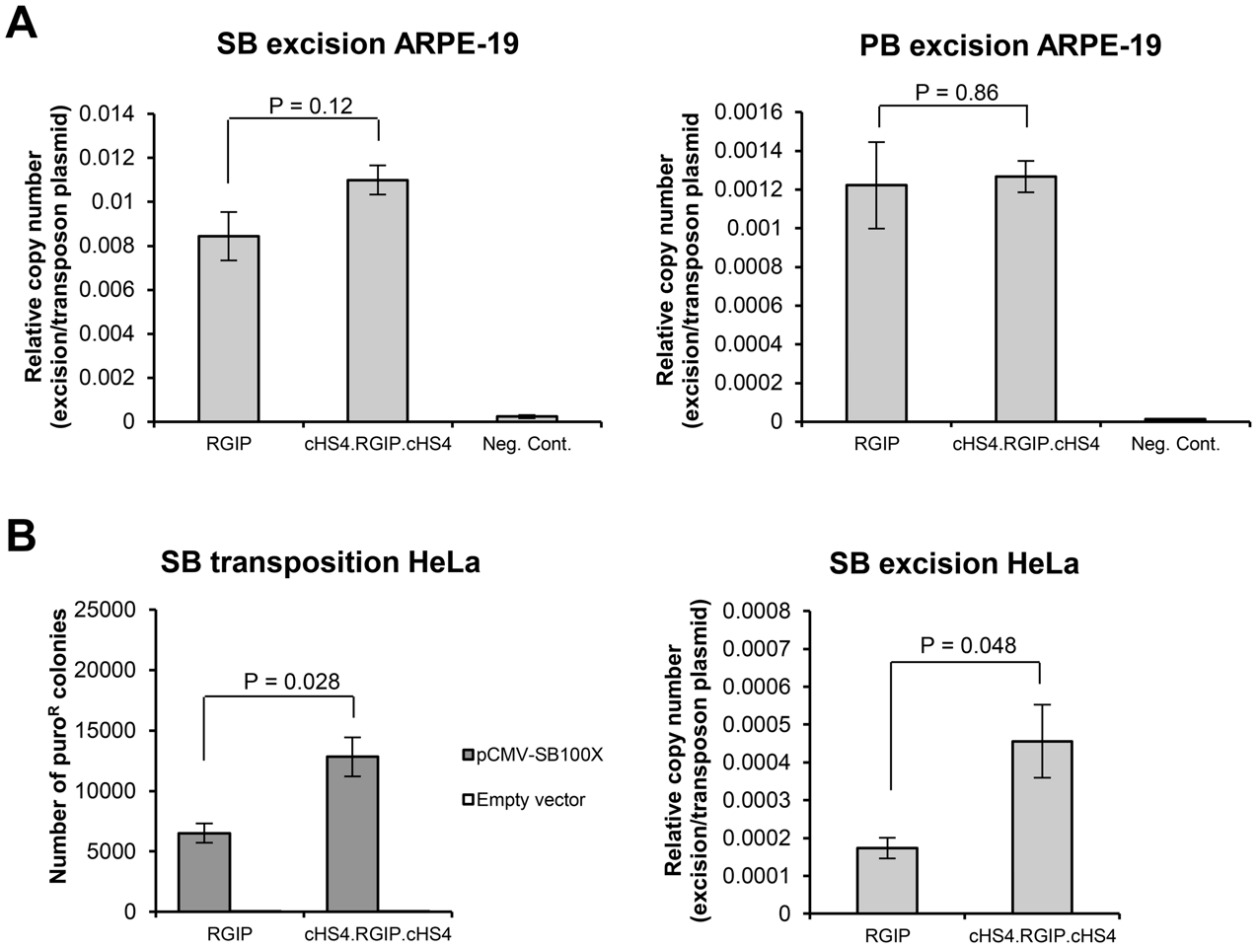
Inclusion of cHS4 sequences results in an improved SB transposon stable transfection rate. Mean ± SEM values are shown (N = 3). P values listed above the brackets were obtained by student's t-tests. (**A**) Measurement of excision circle formation after SB and PB transposition event in ARPE-19 cells. *SB100X* or *iPB* transposase expressing plasmid containing a chloramphenicol resistance gene instead of an ampicillin (Amp) resistance gene was transfected into ARPE-19 cells together with 0.125 pmol of insulated or uninsulated SB or PB transposon plasmid. Transfections of transposon plasmid in the absence of transposase expressing plasmid were included as a negative control. The pBC SK+ plasmid was included as non-specific DNA to ensure that the total amount of DNA was 1 µg for each transfection Two days after transfection, low-molecular weight DNA was extracted, and real-time qPCR analysis was performed using a primer set flanking the transposon excision site. To account for variations in template DNA input, a PCR specific for the Amp gene in the transposon plasmid backbone was utilized to normalize the excision circle data to the amount of transposon plasmid recovered after transfection. (**B**) Stable transfection rate of insulated and uninsulated SB transposons and excision circle formation after SB transposition in HeLa cells. Equal molar amounts of pSBT/RGIP and pSBT/cHS4.RGIP.cHS4 plasmid were cotransfected together with pCMV-SB100X plasmid or pcDNA3.1D/V5.TOPO plasmid (empty vector, only included in transposition assay) into HeLa cells. The number of puromycin-resistant colonies was obtained as described in Figure 1B.

In the context of SB vectors, the stable transfection rate of cHS4-insulated vectors is higher than the uninsulated counterpart (**Figure 3B** and [51]). The number of resistant colonies obtained in colony formation assays is affected by several factors, including transfection efficiency, transposition activity, and expression levels of the resistance gene. To investigate in further detail, if the increase in stable transfection rate observed for the pSBT/cHS4.RGIP.cHS4 vector was due to better transposition of the insulated element, we performed a quantitative PCR assay in ARPE-19 cells to quantify excision circles formed after transposon mobilization from plasmid DNA. A SB100X- or iPB-expressing plasmid, in which the ampicillin (Amp) resistance gene had been replaced by a chloramphenicol resistance gene, was transfected into ARPE-19 cells together with insulated or uninsulated transposon plasmid (equal molar amounts). Transfections of transposon plasmid in the absence of transposase were included as negative controls. Two days after transfection, low-molecular weight DNA was extracted, and real-time qPCR analysis was performed using a primer set flanking the transposon excision site. To account for variations in template DNA input, a PCR specific for the Amp gene in the transposon plasmid backbone was utilized to normalize the excision circle data to the amount of transposon plasmid recovered after transfection. Whereas no difference in the amount of excision circle products was observed for transfections with PBT/RGIP and PBT/cHS4.RGIP.cHS4, a 1.3-fold increase in excision circle formation was observed for transfections with SBT/cHS4.RGIP.cHS4 compared to transfections with SBT/RGIP (**Figure 5A**). Although this increase was not statistically significant (p = 0.12), we reproducibly observed higher levels of excision from the SB vector harboring the insulators, suggesting that the increased stable transfection rate, obtained by flanking insulator sequences in the SB transposon vector, was partly caused by an increased level of transposon mobilization. To validate this finding in another cellular context, we investigated stable transfection rates and plasmid mobilization of SBT/RGIP and SBT/cHS4.RGIP.cHS4 transposons in HeLa cells. As shown in **Figure 5B** (left panel), the stable transfection rate of the insulated SB vector was also increased relative to that of the uninsulated vector in HeLa cells, with a twofold difference between the two transposon constructs (p = 0.028). In the excision assay, a 2.6-fold higher level of excision circle formation was obtained with pSBT/cHS4.RGIP.cHS4 compared to pSBT/RGIP (p = 0.048) (**Figure 5B**, right panel). Collectively, our data demonstrate, that incorporation of cHS4 insulator sequences leads to an increase in the stable transfection rate of SB transposons, and that this increase is most likely caused by a beneficial effect of the cHS4 element on SB transposon mobilization from plasmid DNA as well as on persistency of transgene expression.

## Discussion

In this study we analyzed and compared the transgene expression level from genomically inserted SB, PB, and *Tol2* transposon vectors, encoding an uninsulated or cHS4-insulated RSV-driven eGFP-IRES-puro cassette, in human retinal pigment epithelium cells. By flow cytometric measurements of eGFP expression from single cell clones, we detected transcriptional silencing of the uninsulated transgene cassette in the context of all three vector systems, indicating that transcriptional transgene repression can constitute a problem in ARPE-19 cells. Previous analyses of transgene expression from SB transposon-containing embryonal carcinoma cell clones generated by a nonselective approach showed that a substantial percentage (a minimum of 37%) of the transgene insertions were subjected to complete silencing shortly after integration [51]. In the present study, transposon-containing ARPE-19 clones were generated by the use of an antibiotic selection scheme, which likely leads to a biased isolation of cell clones with transposon insertions situated in more active regions of the chromosome (as opposed to heterochromatin). Still, 55% of the SB clones, 45% of the *Tol2* clones, and 33% of the PB clones had lost more than half of their initial eGFP expression after an 8-week period of growth under nonselective conditions. Histone deacetylation seemed to be part of the transcriptional repression of integrated transgenes, as treatment with the deacetylase inhibitor TSA was observed to regenerate transgene expression. This finding is in agreement with previous analyses of silencing of retroviral [48], [62] and SB vectors [50], [51]. It is likely that other epigenetic mechanisms, such as DNA methylation, were involved in postintegrative gene silencing. Indeed, we have previously observed that addition of the DNA methyltransferase inhibitor 5-Azacytidine could reactivate silenced HeLa and F9 cell clones harboring SB transposon insertions [50], [51].

Extensive silencing of SB transposons carrying RSV-driven eYFP and eGFP expression cassettes has previously been observed in HeLa and F9 cells [50], [51]. In contrast, low levels of transposon vector silencing was observed in a comparison study of gene expression from a Venus-IRES-neo cassette driven by the CAGGS promoter in the context of integrated SB, PB, or *Tol2* transposons in HeLa cells [45]. As transcriptional repression of integrated vectors has been demonstrated to occur in a promoter-dependent manner [50], [63], such discrepancies most likely reflect that the experiments were conducted with transposons containing different cargo sequences, suggesting that silencing of transposon-based vectors can be diminished by careful transgene design. However, substantial differences between cell types may affect the silencing profile. In a recent report examining transgene expression from SB vectors stably transfected into K562 erythroid cells, progressive transgene silencing of the CAGGS promoter was observed and could be significantly reduced by flanking the CAGGS-DsRed or IHK-β-globin gene cassettes with cHS4-insulators [64], indicating that incorporation of protective insulating elements can be beneficial, even when promoter sequences are carefully chosen.

In the present study, all three transposon systems contained the same transgenic cassette, which means that potential intrinsic silencing triggers resident in the RSV promoter or the eGFP gene were present in all vectors. Nevertheless, PB vectors were less affected by transgene silencing compared to vectors based on SB and *Tol2*. Notably, PB has a preference for integrating into transcriptional units, whereas SB has a fairly random integration profile with no preference for or against genes [23], [28], [40], [41], [42], [43]. The integration profile of *Tol2* vectors seems to vary dependent on cell type with a high preference for transcriptional start sites in some cells (HeLa and primary human T cells) and a more random integration pattern in others (zebrafish and HEK293 cells) [43], [44], [45], [46]. Although the integration profile of PB, SB, and *Tol2* vectors has not been examined in ARPE-19 cells, the increased integration frequency of the PB transposon in transcriptionally “open” regions compared to SB and *Tol2* transposons is an obvious explanation for the decreased level of transgene silencing observed for PB. Alternatively, the PB vector may contain protective *cis*-elements in its terminal regions. Analysis of the 5′ and 3′-terminal repeats of the PB transposon has revealed the existence of promoter activity in the 5′-terminal repeat and enhancer activity in the 3′-terminal repeat [65], [66], but the existence of protective motifs has not been reported.

In the attempt to create silencing-protected transposon vectors, we tested the effect of incorporating 1.2-kb cHS4 insulator sequences into the three vector systems, which to our knowledge is the first time the barrier function of the cHS4 element has been tested in PB and *Tol2* transposon vectors. Flanking the transgene cassette with the cHS4 insulator resulted in a 2.2-fold and 1.5-fold increase in the initial eGFP expression level of SB and PB vectors, respectively, despite the fact that the average copy numbers were reduced with the cHS4-containing vectors. Similar results have been observed in studies of transgene expression from retroviral vectors in which cHS4 inclusion led to increased transgene expression levels in transduced murine fibroblast NIH3T3 cell clones [67], [68], human fibrosarcoma HT1080 cell clones [53], and murine primary bone marrow progenitor clones [53]. By comparing the MFI level of stably expressing ARPE-19 clones carrying SB-, PB-, and *Tol2*-derived vectors, we detected large variations of transgene expression within clones containing equal transposon copy numbers, suggesting that eGFP expression levels were not simply determined by transgene copy number, but that chromosomal position effects had a large influence on eGFP expression. Since the cHS4 element does not exhibit conventional enhancer activity, the increase in transgene expression caused by the cHS4 elements likely reflects the ability of the cHS4 insulator to reduce the impact of repressive chromosomal position effects on vector transgene expression. It remains to be elucidated, however, why inclusion of cHS4 sequences into the *Tol2* vector did not seem to benefit the eGFP expression level.

By measuring eGFP expression levels of stably expressing ARPE-19 clones grown for 8 weeks in the absence of selection, we discovered that the cHS4 insulator did not protect against progressive transgene silencing of SB, PB, and Tol2 transposon vectors in ARPE-19 cells. Our previous eGFP expression studies of F9 cell clones harboring the SBT/RGIP or SBT/cHS4.RGIP.cHS4 transposon vector showed that inclusion of cHS4 sequences into the SB transposon had a profound effect on the stability of transgene expression in F9 cell clones after prolonged passaging in culture. Since identical SB transposon constructs were used in this study, differences between cell types most likely accounted for the variable benefit of including cHS4 insulators. In support of this notion, Rivella *et al.* observed that inclusion of the cHS4 insulator into a recombinant retroviral vector could decrease vector methylation and transgene silencing in murine erythroleukemia cells, but not in murine embryonic stem cells [54], indicating that the barrier function of the cHS4 insulator is not uniformly active in all kinds of cell types. To improve the protection against progressive silencing of DNA transposon-embedded transgenes in ARPE-19 cells, the ubiquitously-acting chromatin opening element (UCOE) derived from the human HNRPA2B1-CBX3 locus [69] may represent an attractive alternative to the cHS4 insulator. The UCOE sequence contains a methylation-free CpG island which has been found to shield flanking heterologous promoters from transcriptional silencing, allowing sustained transgene expression from lentiviral vectors [70], [71], [72]. However, this element remains to be investigated in conjunction with a series of promoters and in the context of DNA transposon-based vectors.

Previous studies have determined an inverse relationship between transposon length and transposition frequency for SB transposon vectors [36], [37]. In the present study, we did not observe a reduction in transposition activity when two 1.2-kb cHS4 insulator sequences were incorporated into the pSBT/RGIP vector. Instead, significantly increased stable transfection rates were observed for the insulated SB vector in both ARPE-19 and HeLa cells. By quantitative measurements of SB excision circle formation, we detected a positive effect of the cHS4 sequences on SB transposon mobilization from plasmid DNA. The mechanisms responsible for insulator function are still poorly understood, but an ability of insulator binding proteins to form closed looped chromatin domains has been proposed as one model for insulator enhancer blocking activity [73]. The DNA-bending protein HMGBI is a cofactor of SB transposition in mammalian cells and is believed to stimulate transposition by assisting the SB transposase during synaptic complex formation either by bringing the transposon binding sites and/or the terminal repeats physically closer to each other [74]. Hypothetically, cHS4 insulator binding proteins may also stimulate DNA bending and bring the transposon binding sites closer to each other by loop formation of DNA sequences between the cHS4 element sequences, resulting in increased stabilization of the synaptic complex and increased transposition activities. This hypothesis, however, remains to be tested.

Genotoxicity caused by activation of proto-oncogenes near the vector integration site constitutes a serious problem to gene therapy. As none of the SB, PB, or *Tol2* transposon vectors integrate in a site-specific manner, a risk of insertional mutagenesis upon vector integration exists, a risk especially pronounced for PB and *Tol2* transposons due to their increased preference for integrating into transcriptional units. The cHS4 element contains enhancer blocking activity which enables it to block molecular communication between enhancers and genes. Inclusion of the cHS4 insulator in retroviral vectors has been reported to reduce genotoxicity by 6-fold in a murine tumor transplantation model [75]. The enhancer blocking activity of the cHS4 insulator was not addressed in this study, but previous analysis of promoter activation by an SV40-neo transgene unit within an SB transposon vector showed that the cHS4 element could effectively block transactivation of a nearby TATA-box minimal promoter in HeLa and primary human T cells [76]. Incorporation of cHS4 sequences could therefore potentially lead to an increased safety profile.

By following the expression of a silencing-prone promoter, we demonstrate in this study that the extent of silencing may depend on the type of carrier, most likely due to overall differences in the integration profile of the different DNA transposon carriers. We show that incorporation of cHS4 insulator sequences can lead to an increase in transgene expression levels for genomically integrated SB- and PB-based vectors in ARPE19 cells. In addition, improved stable transfection rates are obtained for cHS4-insulated SB vectors, possibly due to the increased mobilization of cHS4-containing transposons from plasmid DNA. Finally, we find that inclusion of cHS4 elements in SB-, PB- and Tol2-derived vectors does not lead to long-term protection against progressive transgene silencing in ARPE19 cells, supporting the notion that the barrier activity of the cHS4 insulator is not uniformly active in all cell types.

## Materials and Methods

### Plasmid construction

The plasmids pSBT/RGIP, pSBT/cHS4.RGIP.cHS4, and pCMV-SB100X have been described previously [51], [77]. The pPBT/RGIP and pTol2T/RGIP plasmids were constructed by ligation of a RSV.eGFP.IRES.puro PCR fragment, amplified from pSBT/RGIP, into ClaI/NotI-digested pXL-BacII [78] and NheI/ClaI-digested pT2AL200R150 [20], respectively. To generate pPBT/cHS4.RGIP.cHS4 and pTol2T/cHS4.RGIP.cHS4, the 1200-bp cHS4 insulator element was amplified from pSBT/cHS4.RGIP.cHS4 by PCR and inserted in front of and after the RSV.eGFP.IRES.puro cassette in pPBT/RGIP (using a ClaI and NotI site) and in pTol2T/RGIP (using a NheI and ClaI site), respectively. The pCMV-PB and pCMV-Tol2 plasmids have been previously described in [79]. The pCMV-SB100X.chloramp and pCMV-PB.chloramp plasmids were generated by ligation of a chloramphenicol PCR fragment amplified from pBC SK+ (Stratagene, Santa Clara, CA) into PvuI-digested pCMV-SB100X and pCMV-PB, respectively. To generate pPBT÷tp, the transposon sequence of pPBT/RGIP was cut out by NsiI/PstI-digestion, and the digested plasmid backbone was then exposed to T4 DNA polymerase treatment and blunt-end ligation. All produced DNA constructs were verified by restriction digestion and DNA sequencing.

### Cell culture and transposition assays

HeLa (human cervical cancer) cells were maintained in Dulbecco's modified Eagle's medium (DMEM) (Lonza, Basel, Switzerland) supplemented with 10% fetal bovine serum (Lonza, Basel, Switzerland), 0.26 mg/ml glutamine, 54 ng/ml penicillin and 36 ug/ml streptomycin. ARPE-19 human retinal pigment epithelium cells [58] were maintained in culture medium containing 50% Ham's F-12 Nutrient Mixture (Invitrogen, Carlsbad, CA) and 50% DMEM with serum, glutamine, penicillin, and streptomycin, as described above. To measure rates of stable transfection, cells were plated at 1.5 x 10^5^ cells/well in 6-well dishes 1 day before cotransfection with 0.125 pmol transposon plasmid and 0.016 pmol transposase plasmid or pcDNA3.1D/V5.TOPO plasmid (Invitrogen, Carlsbad, CA) as a negative control. The pcDNA3.1D/V5.TOPO plasmid was used as stuffer DNA to obtain equal DNA amounts in each transfection. Transfections were carried out using FuGene-6 (Roche, Basel, Switzerland) according to manufacturer's instructions using 3 µl of reagent per 1 µg of DNA. One day after transfection, cells were split in varying densities and plated in 10-cm dishes. Two days after transfection, selection medium containing 1 µg/ml puromycin (Invitrogen, Carlsbad, CA) was added to the cells. After 8 days of selection, colonies of cells were stained with 0.6% methylene blue, air-dried and counted.

### Generation of stable expressing cell clones and long-term expression analysis

ARPE-19 cells were seeded in 6-well dishes (1.5×10^5^ cells/well) and transfected with 0.125 pmol transposon plasmid together with 0.05 µg transposase plasmid using FuGene-6 transfection reagent according to manufacturer's instructions. One day after transfection cells were split in varying densities and plated in 10-cm dishes. Selection medium, containing 1 µg/ml puromycin, was added to the cells two days after transfection. After 10 days of selection, single clones were isolated and expanded for genomic DNA extraction and long-term eGFP expression analysis. The isolated, stably expressing cell clones were passaged for 8 weeks in standard culture medium, and analyzed by flow cytometry on day 0 and day 56 on a BD FACSAria III cell sorter (BD Biosciences, San Jose, CA). In the flow cytometric analysis, non-transfected cells were included as a negative control, and propidium iodide (Sigma Aldrich, St Louis, MO) was used to exclude non-viable cells.

### Transposon excision assay

HeLa or ARPE-19 cells were seeded in 6-well dishes (1.5×10^5^ cells/well) and transfected with 0.25 pmol (HeLa) or 0.125 pmol (ARPE-19) transposon plasmid together with 0.25 pmol (HeLa) or 0.05 µg (ARPE-19) transposase plasmid or pBC SK+ plasmid as negative control. The pBC SK+ plasmid was also used as stuffer DNA. Transfections were carried out using FuGene-6 according to manufacturer's instructions using 3 µl of reagent per 1 µg of DNA. Low-molecular weight DNA was extracted 2 days after transfection using the QIAprep miniprep kit (CA 91355; Qiagen, Valencia, Spain) according to the manufacturer's instructions, except for a 1-h incubation period at 55°C (0.6% SDS and 0.08 mg/ml proteinase K together with buffer P1) instead of the lysis step with buffer P2 after resuspension of the cell pellet. 50 ng of extracted DNA from each transfection was used in a quantitative PCR analysis using a primer set recognizing transposon excision circle products and a primer set amplifying an amplicon within the Amp resistance gene. The plasmids pUC19 and pPBT÷tp were used as templates for standard curve formation. The analysis was performed on a Lightcycler 480 (Roche, Basel, Switzerland) using the Lightcycler DNA Master SYBR Green I kit (Roche, Basel, Switzerland). Amplification was performed under the following conditions: Thirty cycles (95°C 30s, 65°C 30s, and 72°C 5s). Primer sequences are as follows: SB excision (S): 5′-CGATTAAGTTGGGTAACGCCAGGG-3′, SB excision (AS): 5′-CAGCTGGCACGACAGGTTTCCCG-3′, PB excision (S): 5′-CCGTGGAGGACGGGCAGACTCGCG-3′, PB excision (AS): 5′-GGCGTGCATGGCCACACCTTCCCG-3′, Amp (S): 5′-CAAGAGCAACTCGGTCGC-3′, Amp (AS): 5′-TCGTTGTCAGAAGTAAGTTGGC-3′.

### Southern Blot analysis

Genomic DNA was prepared from cell pellets following NaCl extraction and ethanol precipitation. 15 μg genomic DNA was digested with PstI+KpnI (pSBT/RGIP stable clones), KpnI+SalI (pSBT/cHS4.RGIP.cHS4 stable clones), KpnI+PvuII (pPBT/RGIP stable clones) or KpnI (pTol2T/RGIP, pPBT/cHS4.RGIP.cHS4, and pTol2T/cHS4.RGIP.cHS4 stable clones). Genomic DNA of non-transfected cells was used as a negative control. Genomic DNA of non-transfected cells spiked with plasmid DNA corresponding to 1 copy/cell or 3 copies/cell was used as a positive control. The digested DNA was electrophoresed in a 0.8% agarose gel and transferred to a Hybond membrane (GE Healthcare, Buckinghamshire, UK). The membrane was hybridized overnight using a puro-specific [α-32P] dCTP-labelled probe.

### TSA treatment

Silenced ARPE-19 cell clones were grown in the presence of 1200 nmol/l TSA (Sigma Aldrich, St Louis, MO). The clones were treated 24 hours before analysis of eGFP expression by flow cytometry on a BD FACSAria III cell sorter (BD Biosciences, San Jose, CA). Non-transfected cells were included as a negative control, and propidium iodide (Sigma Aldrich, St Louis, MO) was used to exclude non-viable cells.

## Supporting Information

Figure S1 **eGFP expression profiles of pSBT/RGIP clones measured by flow cytometry at day 0 and day 56 of growth in non-selection medium.**
(PDF)

Figure S2 **eGFP expression profiles of pPBT/RGIP clones measured by flow cytometry at day 0 and day 56 of growth in non-selection medium.**
(PDF)

Figure S3 **eGFP expression profiles of pTol2/RGIP clones measured by flow cytometry at day 0 and day 56 of growth in non-selection medium.**
(PDF)

Figure S4 **eGFP expression profiles of pSBT/cHS4.RGIP.cHS4 clones measured by flow cytometry at day 0 and day 56 of growth in non-selection medium.**
(PDF)

Figure S5 **eGFP expression profiles of pPBT/cHS4.RGIP.cHS4 clones measured by flow cytometry at day 0 and day 56 of growth in non-selection medium.**
(PDF)

Figure S6 **eGFP expression profiles of pTol2T/cHS4.RGIP.cHS4 clones measured by flow cytometry at day 0 and day 56 of growth in non-selection medium.**
(PDF)

Figure S7 **Median fluorescence intensity** (**MFI**) **values of RPE transposon clones measured by flow cytometry at day 0 of passage.**
(PDF)
