# Supplementary figures and images for: The Impact of cHS4 Insulators on DNA Transposon Vector Mobilization and Silencing in Retinal Pigment Epithelium Cells
Source: PLoS One. 2012 Oct 26;7(10):e48421. doi: 10.1371/journal.pone.0048421 (PMC3482222; doi:10.1371/journal.pone.0048421)

Sharma et al., Supplementary Figure S1  
SBT/RGIP clones

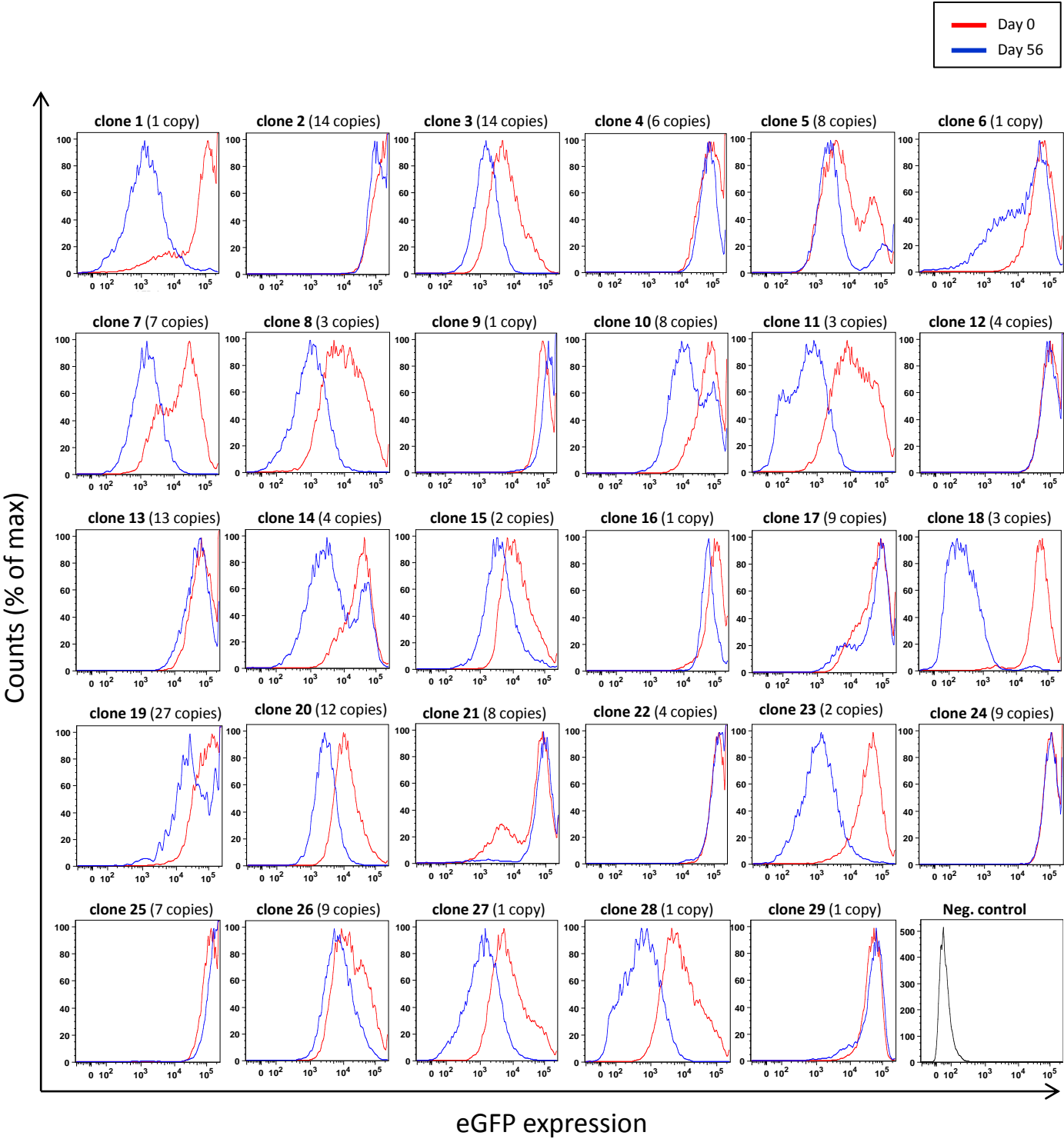

Supplement: Figure S1 — eGFP expression profiles of pSBT/RGIP clones measured by flow cytometry at day 0 and day 56 of growth in non-selection medium. (PDF) [file pone.0048421.s001.pdf]

Sharma et al., Supplementary Figure S2  
PBT/RGIP clones

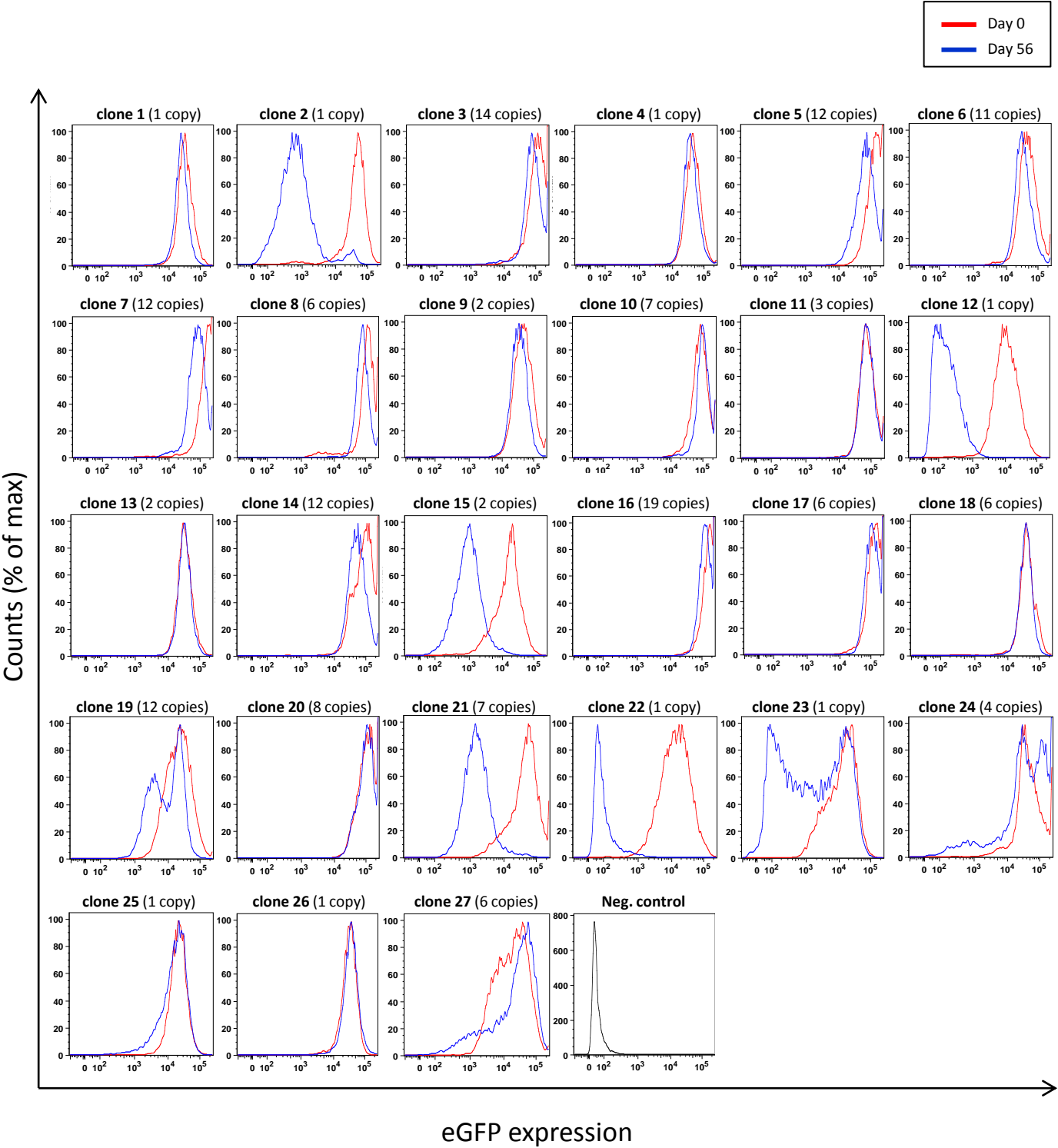

Supplement: Figure S2 — eGFP expression profiles of pPBT/RGIP clones measured by flow cytometry at day 0 and day 56 of growth in non-selection medium. (PDF) [file pone.0048421.s002.pdf]

Sharma et al., Supplementary Figure S3  
Tol2T/RGIP clones

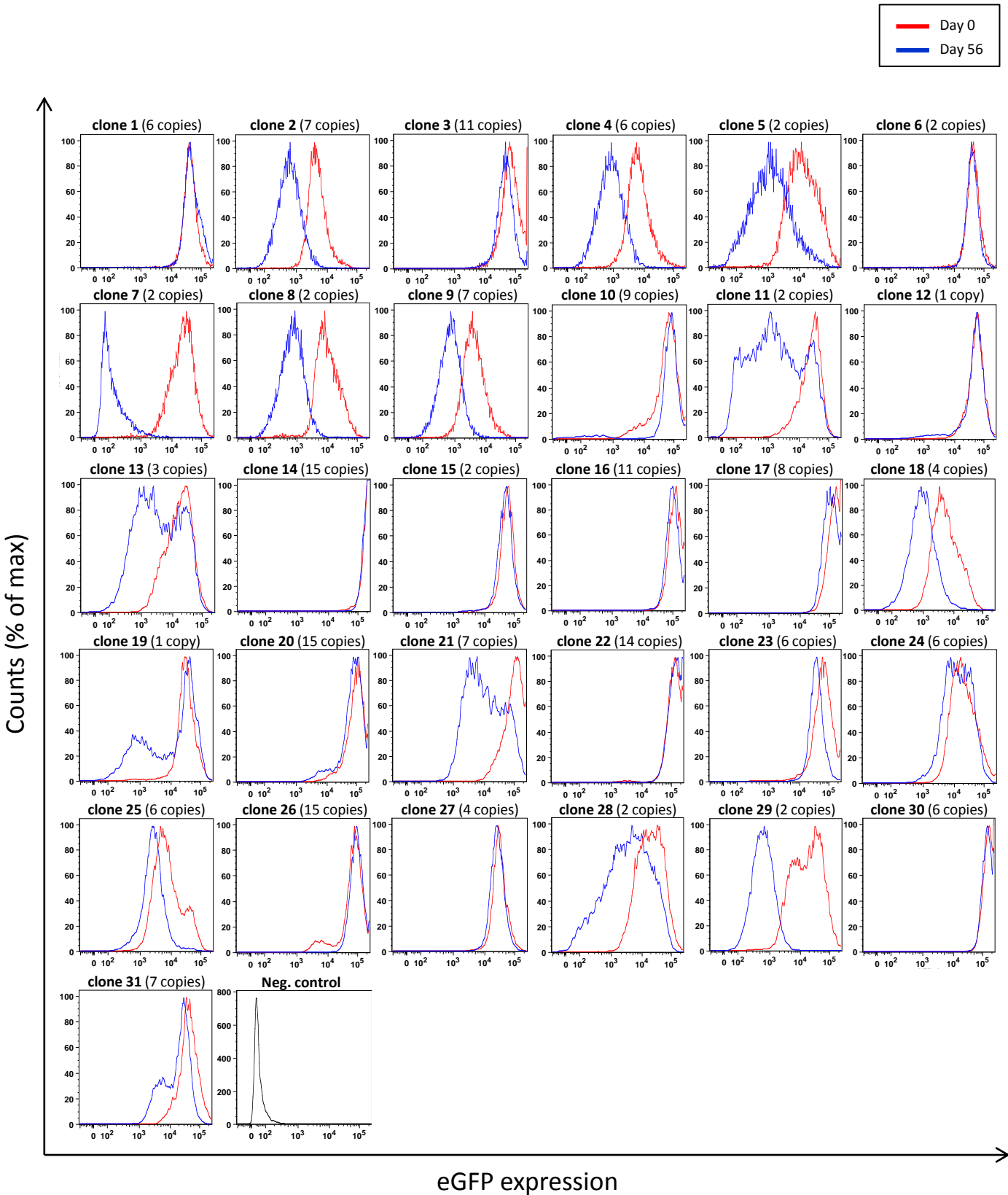

Supplement: Figure S3 — eGFP expression profiles of pTol2/RGIP clones measured by flow cytometry at day 0 and day 56 of growth in non-selection medium. (PDF) [file pone.0048421.s003.pdf]

Sharma et al., Supplementary Figure S4

SBT/cHS4.RGIP.cHS4 clones

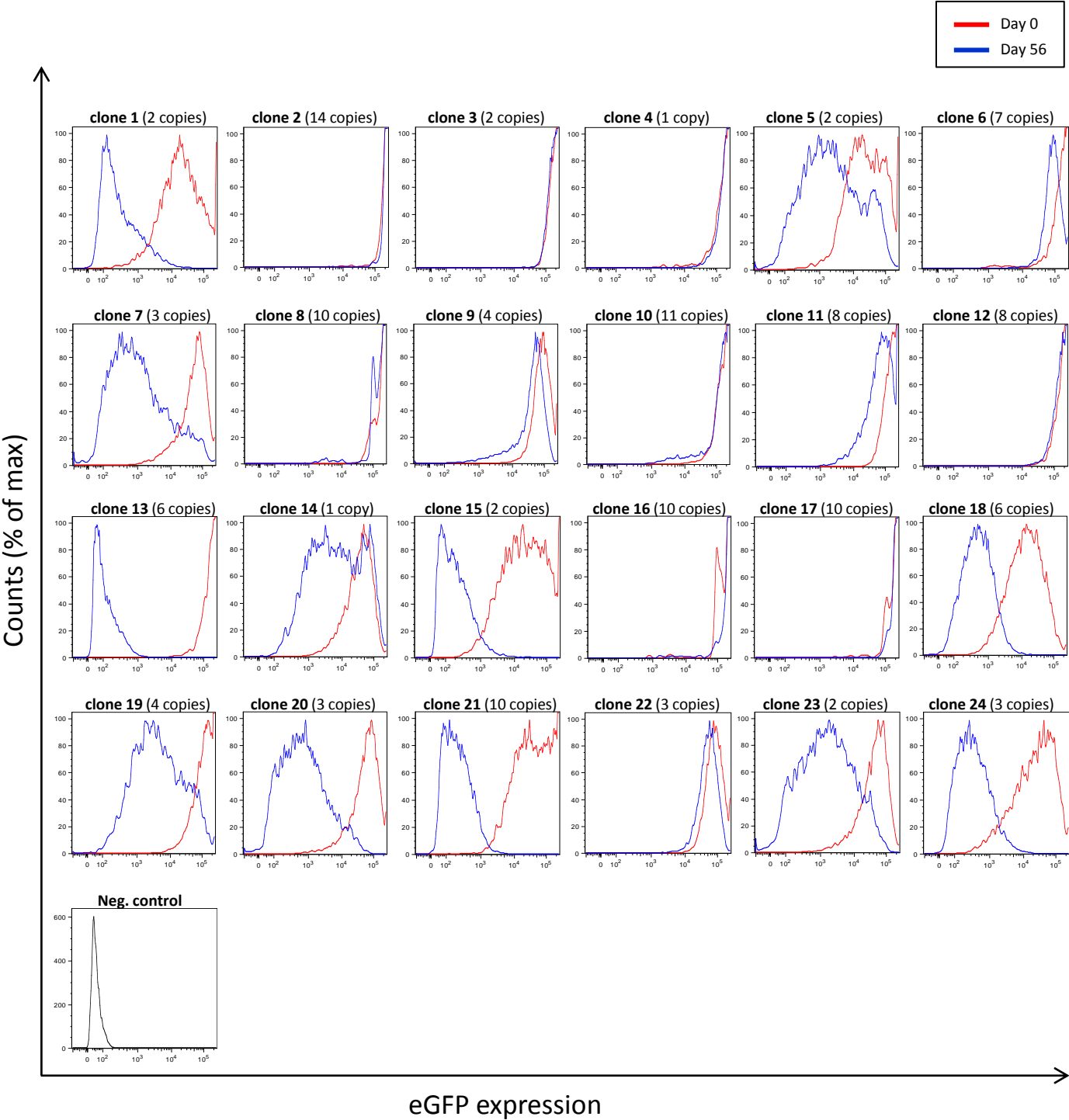

Supplement: Figure S4 — eGFP expression profiles of pSBT/cHS4.RGIP.cHS4 clones measured by flow cytometry at day 0 and day 56 of growth in non-selection medium. (PDF) [file pone.0048421.s004.pdf]

Sharma et al., Supplementary Figure S5  
PBT/cHS4.RGIP.cHS4 clones

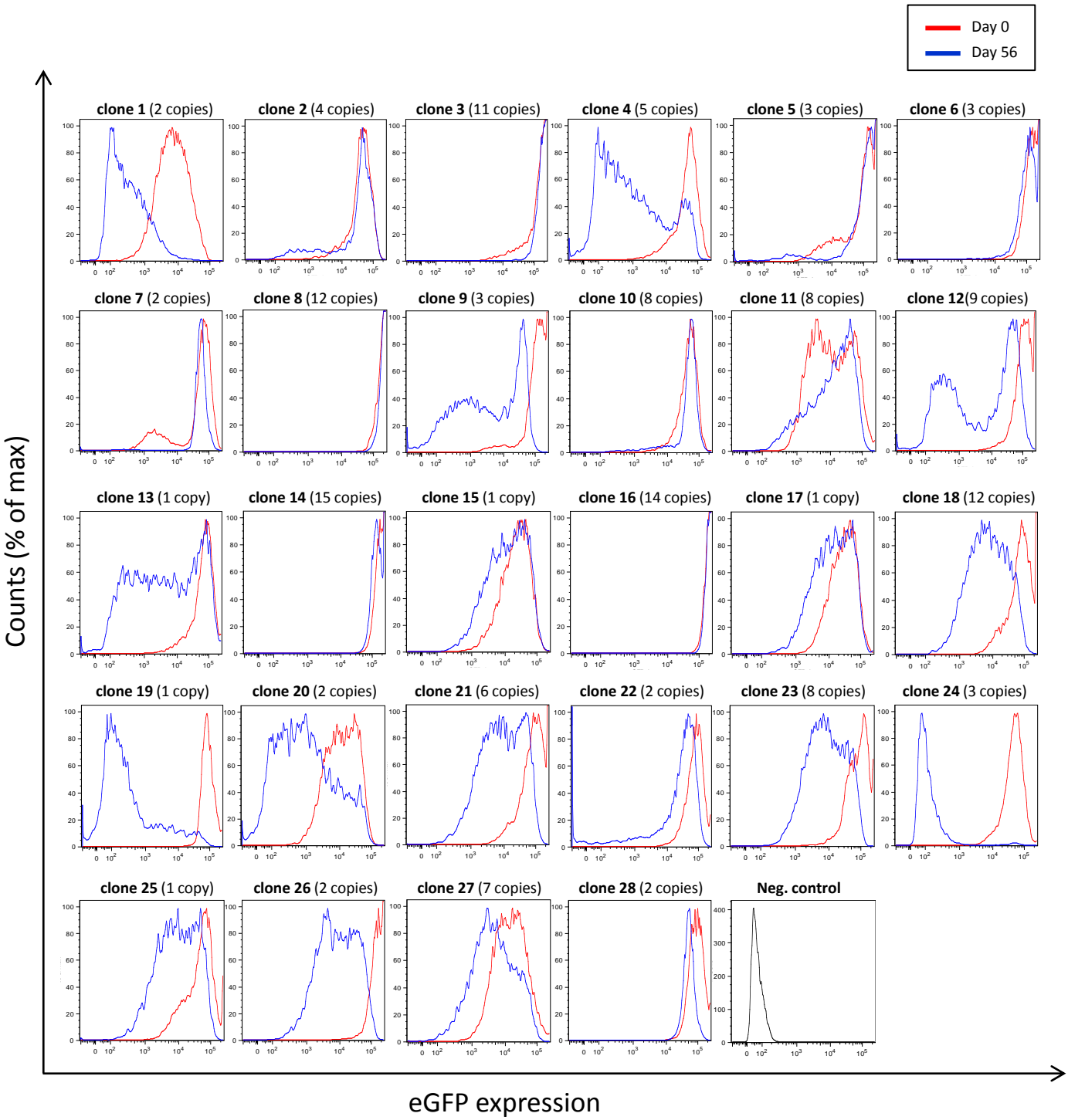

Supplement: Figure S5 — eGFP expression profiles of pPBT/cHS4.RGIP.cHS4 clones measured by flow cytometry at day 0 and day 56 of growth in non-selection medium. (PDF) [file pone.0048421.s005.pdf]

Sharma et al., Supplementary Figure S6  
Tol2T/cHS4.RGIP.cHS4 clones

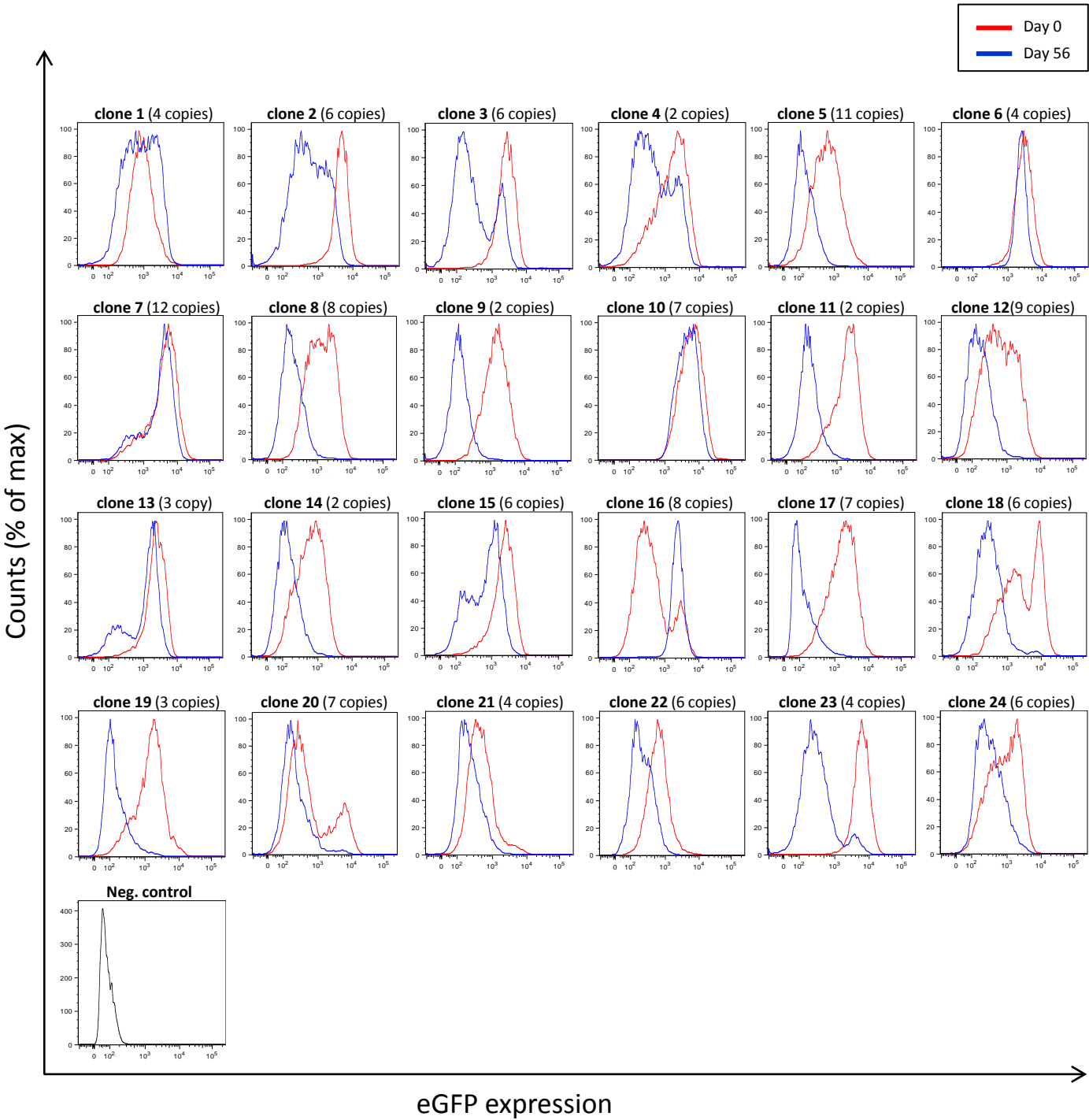

Supplement: Figure S6 — eGFP expression profiles of pTol2T/cHS4.RGIP.cHS4 clones measured by flow cytometry at day 0 and day 56 of growth in non-selection medium. (PDF) [file pone.0048421.s006.pdf]

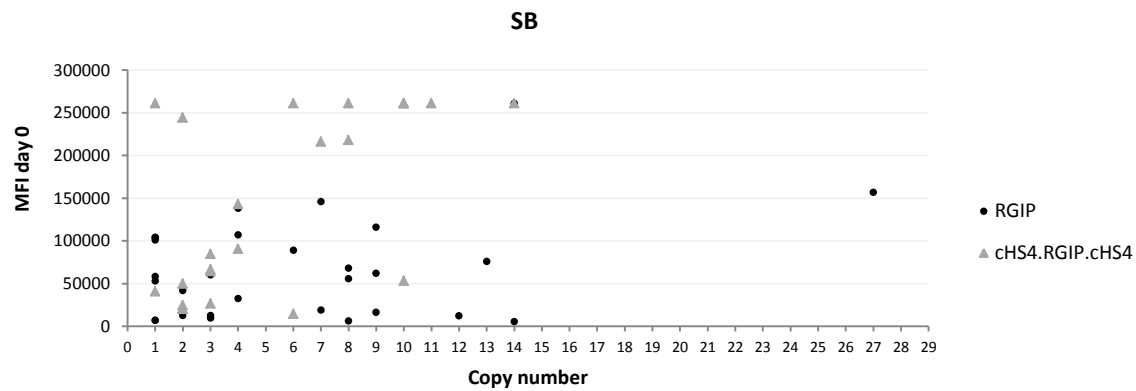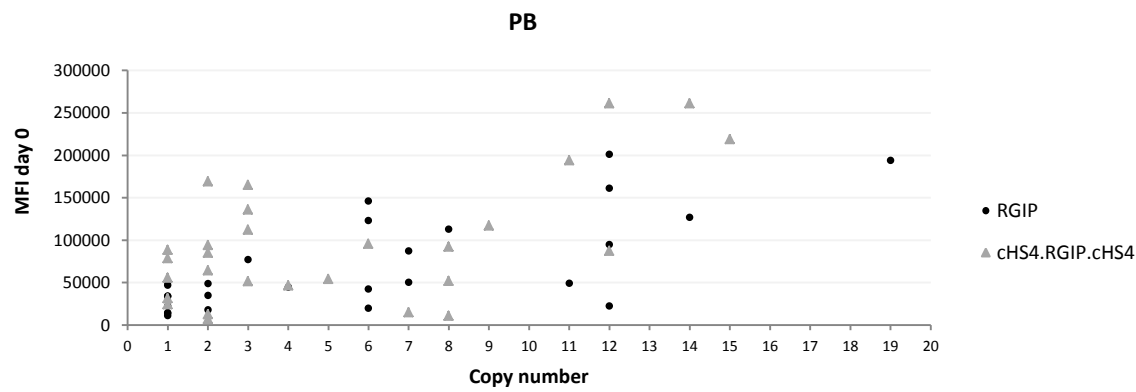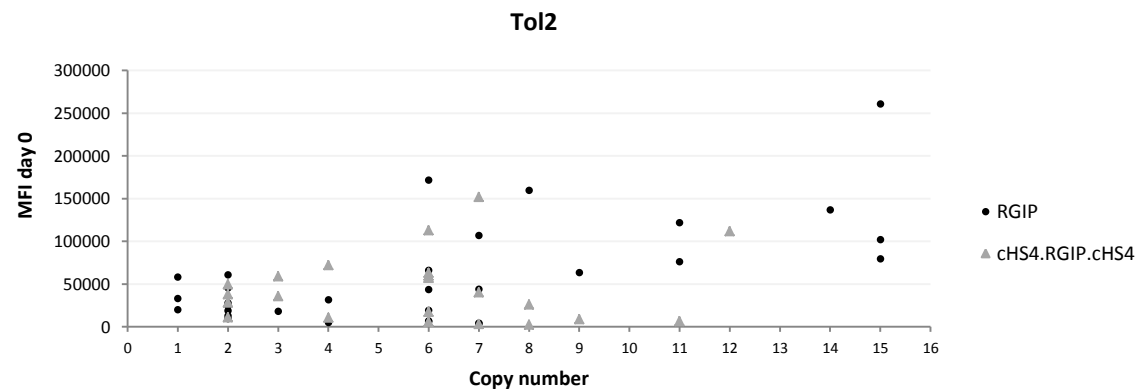

Supplement: Figure S7 — Median fluorescence intensity (MFI) values of RPE transposon clones measured by flow cytometry at day 0 of passage. (PDF) [file pone.0048421.s007.pdf]
